# JoGH policy on the use of artificial intelligence in scholarly manuscripts

**DOI:** 10.7189/jogh.13.01002

**Published:** 2023-02-03

**Authors:** Ana Marušić

**Affiliations:** Co-editor in Chief, *Journal of Global Health*

We witness the increasing use of artificial intelligence (AI) in science, and the recent release of the ChatGPT chatbot has brought generative AI into publishing.

A chatbot is defined as “a computer program that simulates and processes human conversation (either written or spoken), allowing humans to interact with digital devices as if they were communicating with a real person” [1].

Since the introduction of ChatGPT by OpenAI in November 2022 [2], journals have published articles with ChatGPT as authors. To address this issue, the *Journal of Global Health* adopts the recommendations from the World Association of Medical Editors (WAME) to update our policy on authorship [3]:

1. Chatbots cannot be authors.

Chatbots cannot meet the requirements for authorship as they cannot understand the role of authors or take responsibility for the paper. Chatbots cannot meet the authorship criteria of the International Committee of Medical Journal Editors (ICMJE) [4], particularly the requirements for final approval of the manuscripts and taking accountability for the work. A chatbot cannot understand a conflict of interest statement, or have the legal standing to sign a statement. Chatbots have no affiliation independent of their creators. They cannot hold copyright. Authors submitting a manuscript must ensure that all those named as authors meet the authorship criteria, which clearly means that chatbots should not be included as authors.

2. Authors should be transparent when chatbots are used and provide information about how they were used.

Since the field is evolving quickly at present, authors using a chatbot to help them write a paper should declare this fact and provide full technical specifications of the chatbot used (name, version, model, source) and method of application in the paper they are submitting (query structure, syntax). This is consistent with the ICMJE recommendation of acknowledging writing assistance [4].

3. Authors are responsible for the work performed by a chatbot in their paper (including the accuracy of what is presented, and the absence of plagiarism) and for appropriate attribution of all sources (including for material produced by the chatbot).

Human authors of articles written with the help of a chatbot are responsible for the contributions made by chatbots, including their accuracy. They must be able to assert that there is no plagiarism in their paper, including in text produced by the chatbot. Human authors must ensure there is appropriate attribution of all quoted material, including full citations. They should declare the specific query function used with the chatbot. Authors will need to seek and cite the sources that support the chatbot’s statements. Since a chatbot may be designed to omit sources that oppose viewpoints expressed in its output, it is the authors’ duty to find, review and include such counterviews in their articles.

In line with the 4th WAME recommendation [3], we will use manuscript evaluation tools when they become available to detect AI use in text and image creation.

I would like to use this opportunity to inform our readers that I will be stepping down as the Co-editor in Chief of the *Journal of Global Health*. After full twelve great years of working with outstanding experts in global health and working with you on creating the journal, I am ready to take new challenges, just as we all face new challenges in scientific publishing.
